# Supplementary material for: SynVerse: a modular framework for building and evaluating deep learning-based drug synergy prediction models
Source: Brief Bioinform. 2025 Dec 31;26(6):bbaf676. doi: 10.1093/bib/bbaf676 (PMC12753315; doi:10.1093/bib/bbaf676)
Supplement: Supplementary_Notes_bbaf676 [file supplementary_notes_bbaf676.pdf]

SynVerse: A Modular Framework for Building and Evaluating Deep Learning Based Drug Synergy Prediction Models

Nure Tasnina, Maryam Haghani, and T. M. Murali*

Department of Computer Science, Virginia Tech, Blacksburg, VA 24060, USA

1 Supplementary Notes

1.1 Hyperparameter Tuning

We used BOHB [1], a robust hyperparameter optimization method, to navigate the extensive hyperparameter space of our models, which included architectural parameters (e.g., the number and width of layers), optimization parameters (e.g., learning rate), and regularization parameters (e.g., dropout rates) (Supplementary Table 1). BOHB efficiently identifies high-performing configurations by combining the rapid exploration capabilities of HyperBand [2] with the convergence guarantees offered by Bayesian Optimization [3]. Instead of relying on random sampling, BOHB incorporates a Bayesian optimization component to guide the search effectively. For our experiments, we utilized the Python library HpBandSter, developed by the authors of BOHB.

We performed hyperparameter tuning using a validation set comprising 25% of the training triplets. We applied early stopping with a patience of 90 epochs. We also used an adaptive learning rate strategy, ReduceLROnPlateau, which decreased the learning rate by a factor of 0.5 if the validation loss did not improve for 20 consecutive epochs.

*Corresponding author: murali@cs.vt.edu

Model Components	Parameter	Range of values
MLP	Learning rate	$[10^{-5}, 10^{-3}]$
	Optimizer	{Adam, SGD}
	SGD Momentum	[0.5, 0.99]
	Number of hidden layers	{1, 2, 3}
	First layer	{128, 256, 512, 1024, 2048, 4096}
	Second layer	{128, 256, 512, 1024, 2048, 4096}
	Third layer	{128, 256, 512, 1024, 2048, 4096}
	Dropout rate at input layer	[0, 0.5]
	Dropout rate at hidden layer	[0, 0.5]
GCN	Number of convolution layer	{1, 2, 3}
	First convolution layer	{256, 512, 1024, 2048, 4096}
	Second convolution layer	{256, 512, 1024, 2048, 4096}
	Third convolution layer	{256, 512, 1024, 2048, 4096}
	Number of feed forward layers	{1, 2, 3}
	First feed forward layer	{256, 512, 1024, 2048, 4096}
	Second feed forward layer	{256, 512, 1024, 2048, 4096}
	Third feed forward layer	{256, 512, 1024, 2048, 4096}
	Dropout rate	[0, 0.5]
	Batch nomalization	{True, False}
Transformer	Number of transformer layers	{2, 3, 4}
	Embedding dimension	{64, 128, 256, 512, 1024}
	Number of heads	{4, 8}
	Dimension of feed forward layer	{64, 128, 256, 512, 1024}
	Maximum sequence length	320
	Positional encoding type	{'learnable', 'fixed'}
	Batch nomalization	{True, False}

Supplementary Table 1: **Hyperparameter configuration explored by BOHB for different models.**

1.2 Dataset Statistics: S_{mean} Score

To facilitate the training and evaluation of models using different features, we constructed three subsets of triplets from DrugComb. The first subset included only those triplets for which SMILES strings (or features derived from them) were available for both drugs. The second subset comprised triplets with drug target information. The third subset contained triplets with gene expression data available for the corresponding cell lines. We imposed a minimum threshold of 5% on the abundance of triplets per cell line in the final dataset used for SynVerse, allowing different cell lines to qualify for inclusion across subsets.

SMILES-based subset contained 105, 707 triplets, 2, 582 drugs, and 9 cell lines; the target-based subset included 53, 843 triplets, 275 drugs, and 13 cell lines; and the gene expression-based subset comprised 104, 082 triplets, 194 drugs, and 15 cell lines. In the SMILES-based subset, the cell line *kbm7* was the most represented, appearing in 38, 227 triplets (36% of the subset), whereas *wm115* had the fewest, with 5, 365 triplets (5%) (Supplementary Figure 1A). The S_{mean} score in this subset had a mean of 15.01 and a standard deviation of 29.27 (Supplementary Figure 1B).

Within the drug target-based subset, *kbm7* again had the largest presence, contributing 15, 225 triplets (28%), while *istmel1* was the least represented with 2, 775 triplets (5%) (Supplementary Figure 1A). This subset showed a similar distribution of synergy scores, with a mean of 15.76 and a standard deviation of 29.23 (Supplementary Figure 1B).

In contrast, the gene expression-based subset had a different distribution: *uacc257* contributed the highest number of triplets at 9, 618 (9%), and *melho* had the lowest with 5, 356 (5%) (Supplementary Figure 1A). This group displayed a slightly higher average synergy score of 17.18, accompanied by a larger standard deviation of 36.25 (Supplementary Figure 1B).

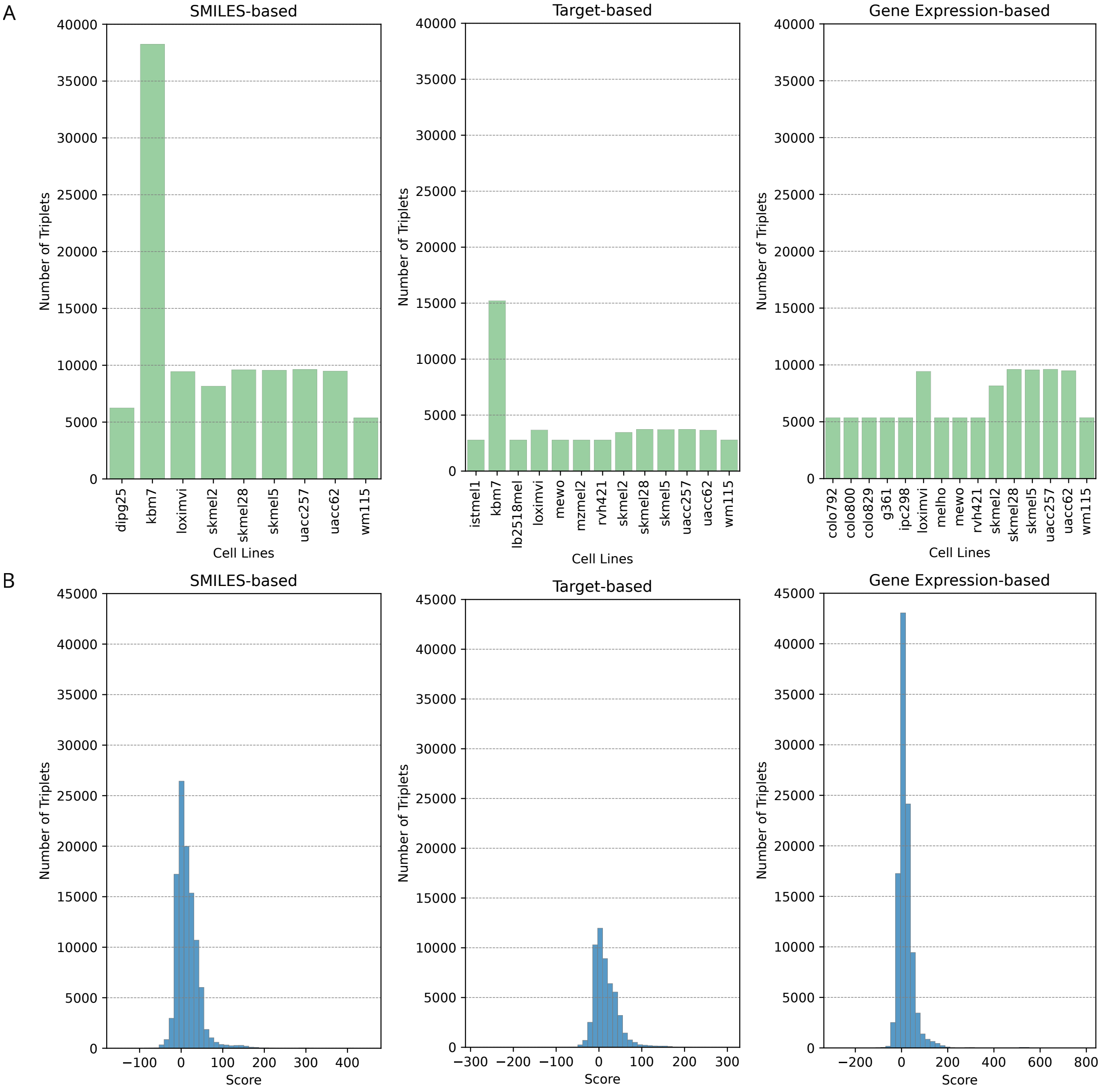

Supplementary Figure 1: **Distribution of S_{mean} score in the DrugComb dataset**. **A.** The x-axis shows the names of cell lines, and the y-axis indicates the number of drug-drug-cell line triplets associated with each cell line. **B.** The x-axis displays binned S_{mean} synergy scores, and the y-axis shows the number of triplets falling within each score bin.

1.3 Evaluated Models

A user of SynVerse has the flexibility to choose any combination of drug and/or cell line features along with compatible preprocessing techniques and encoders implemented in SynVerse. For our analysis, though, we focused on the subset of models that used only one feature, optionally applying a preprocessing step and a single encoder to generate the final embedding (Supplementary Table 2).

**Model name**	**Drug feature**			**Cell line feature**		
	**Feature name**	**Preprocessing**	**Encoder**	**Feature name**	**Preprocessing**	**Encoder**
MACCS	MACCS	-	-	One hot	-	-
MACCS (AE)	MACCS	Autoencoder, Standardization	-	One hot	Autoencoder, Standardization	-
ECFP	ECFP_4	-	-	One hot	-	-
ECFP (AE)	ECFP_4	Autoencoder, Standardization	-	One hot	Autoencoder, Standardization	-
MFP	Morgan Fingerprint	-	-	One hot	-	-
MFP (AE)	Morgan Fingerprint	Autoencoder, Standardization	-	One hot	Autoencoder, Standardization	-
Mol Graph (GCN)	Molecular structure	-	GCN	One hot	-	-
SMILES (Transformer)	SMILES	-	Transformer	One hot	-	-
SMILES (SPMM)	SMILES	SPMM	-	One hot	-	-
SMILES (KPGT)	SMILES	KPGT	-	One hot	-	-
SMILES (MolE)	SMILES	MolE	-	One hot	-	-
Target	Drug target	-	-	One hot	-	-
Target (RWR)	Drug target	RWR on PPI network	-	One hot	-	-
Target (AE)	Drug target	Autoencoder, Standardization		One hot	Autoencoder, Standardization	-
Genex	One hot	-	-	Gene expression	Standardization	-
LINCS_1000	One hot	-	-	Expression of 978 Landmark genes	Standardization	-
Baseline	One hot	-	-	One hot	-	-

Supplementary Table 2: **Specification of models**. The combination of features, preprocessing techniques, and encoders evaluated by SynVerse.

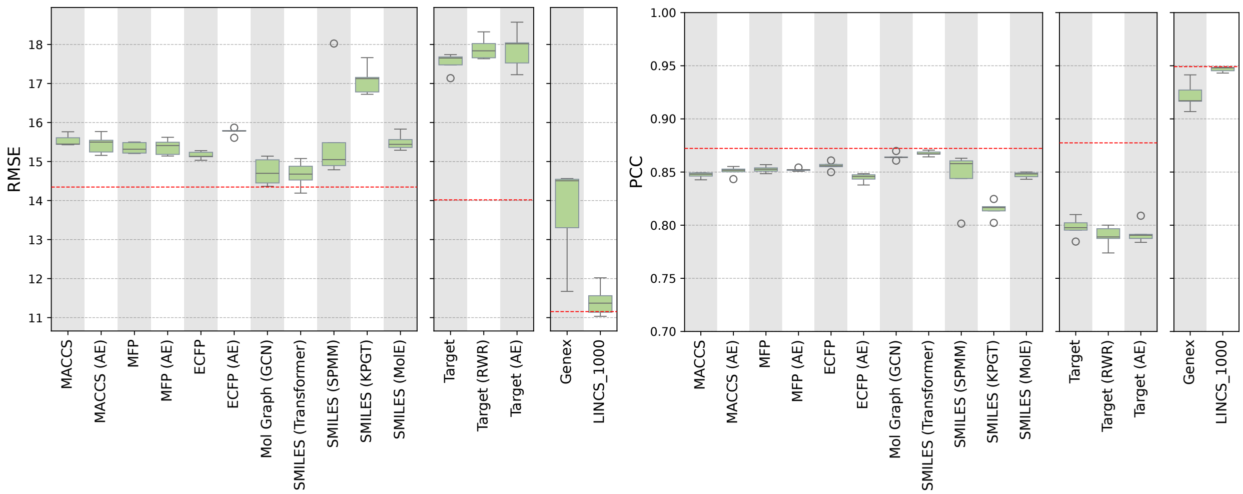

Supplementary Figure 2: **Evaluation of models in predicting S_{mean} score using leave triplet split.** The x-axis represents the models, the y-axis displays RMSE (left panel) and PCC (right) between true and predicted scores by each model across five independent runs. The red dotted line indicates the median performance of the baseline model.

1.4 Performance of Models Predicting S_{mean} Using Leave Triplets Strategy

We aimed to assess the predictive value of individual drug and cell line features for synergy prediction on unseen drug-drug-cell line triplets (Supplementary Figure 2).

Models that used SMILES or SMILES-derived features showed a significant difference in their performance (p-value of 2.3×10^{-5} for RMSE, p-value of 9.51×10^{-6} for PCC; Kruskal-Wallis test). Among them, *Mol Graph (GCN)* and *SMILES (Transformer)* achieved the lowest median RMSE (14.7 for both) and the highest median PCC (0.86 and 0.87, respectively). Both of them performed significantly better than the other models (p-value < 0.02 for PCC, one-sided Mann-Whitney U test with Benjamini-Hochberg correction). There was no significant difference between these two models (p-value of > 0.13 for PCC, two-sided Mann-Whitney U test with Benjamini-Hochberg correction).

The models exploiting drug target based features were similar to each other in performance, with median RMSE ranging from 17.65 to 18.02 and PCC ranging from 0.79 to 0.8 (p-value > 0.27 for RMSE and PCC; Kruskal-Wallis test).

Among the models utilizing gene expression data, *LINCS_1000*, which uses expression from 978 landmark genes, achieved the best performance with a median RMSE of 11.37 and a PCC of 0.95. It significantly outperformed the *Genex* model, which leverages expression data from all available genes (median RMSE of 14.5, PCC of 0.92; adjusted p-value $= 0.004$ for PCC, one-sided Mann-Whitney U test with Benjamini-Hochberg correction).

Overall, the models demonstrated moderate to strong predictive capabilities for unseen triplets. However, none outperformed the baseline model (adjusted p-value of 1.0).

1.5 Performance of Baseline vs. Two State-of-the-art Models on Model-specific Datasets

We did not evaluate any previously published models directly in our main experiments. However, to gauge the performance of our implemented baseline (using only one-hot encoding as features), we trained and tested it on the preprocessed synergy datasets released by two recent models: MARSY [4] and SynergyX [5]. MARSY is a multitask deep learning model that predicts drug synergy and individual drug responses by combining untreated cell line gene expression profiles with drug-induced differential expression signatures. It employs MLP-based encoders for drug pair and drug-cell line features, which it fuses through a final MLP to produce three outputs: responses for each drug and the synergy score. SynergyX, a transformer-based multimodal model, leverages mutual and self-attention modules to capture drug-drug and drug-cell interactions. It represents drugs using SMILES-derived ESPF fingerprints and incorporates six types of cell line features: gene expression, mutations, copy number, methylation, gene effect, and dependency probability.

We used the preprocessed synergy datasets provided by the authors and split each into training, validation, and test sets using a 3:1:1 ratio using the leave drug pair strategy. We tuned our baseline model on the validation set and reported its performance on the test set using the optimal hyperparameters.

Note that we did not rerun MARSY or SynergyX but reported results published by the authors. Although we used the same input data and the same data splitting strategy (i.e., leave drug pair), it is possible that our train-validation-test splits differ from those in the original studies. Nevertheless, the baseline model showed low variation across five independent runs (standard deviations of 0.01 and 0.005 for PCC), indicating that the results remained consistent across different data partitions.

1.6 Performance of Classical Machine Learning Models

We trained two traditional machine learning models, Extreme Gradient Boosting (XGB) [6] and Random Forest (RF) [7], to predict the synergy score on DrugComb dataset using S_{mean} score. We used the same preprocessed synergy dataset (Supplementary Note 1.1) and the same data splits as those used in the DL-based models in SynVerse, to ensure consistency. We used one feature at a time for the drug (or cell line) to train and evaluate the models. We did not include the gene expression feature, Genex in this analysis due to its high dimensionality (20,068 genes) for which tree-based ensemble methods are computationally intensive and scale poorly.

For XGB, we used the XGBRegressor implementation from the `xgboost` python package and performed a grid search over the following hyperparameters for tuning: number of estimators ($n\_estimators$ = 100, 200), maximum tree depth (max_depth = 6, 7, 10), learning rate ($learning\_rate$ = 0.1, 0.2, 0.3), and subsampling ratio ($subsample$ = 0.8, 1.0). For the Random Forest model, we used the RandomForestRegressor from the `scikit-learn` package [8] and optimized its hyperparameters using `RandomizedSearchCV` with 50 iterations to efficiently explore the parameter space. We tuned the following hyperparameters: number of trees ($n\_estimators$= 100, 200, 300, 400, 500, 600), maximum depth of each tree (max_depth: 8, 12, 16, 20, 24, 28, 32), minimum samples required to split a node ($min\_samples\_split$: 2, 3, 4, 5, 6, 7, 8, 9, 10), minimum samples required at each leaf node ($min\_samples\_leaf$: 1, 2, 4, 8), and the number of features considered at each split ($max\_features$: "sqrt", 0.8, 0.5). We enabled out-of-bag (OOB) estimation and used a warm-start strategy to determine the optimal number of trees. We applied early stopping when OOB improvement fell below a predefined tolerance.

Across four data splitting strategies: leave drug pair, leave drug, and leave cell line, and leave triplet, none of XGB or RF significantly outperformed the baseline (adjusted p-value>= 0.35; one-sided Mann-Whitney U test with Benjamini-Hochberg correction) (leave drug pair split in Supplementary Figure 3; leave drug, leave cell line, and leave triplet split in Supplementary Figure 4).

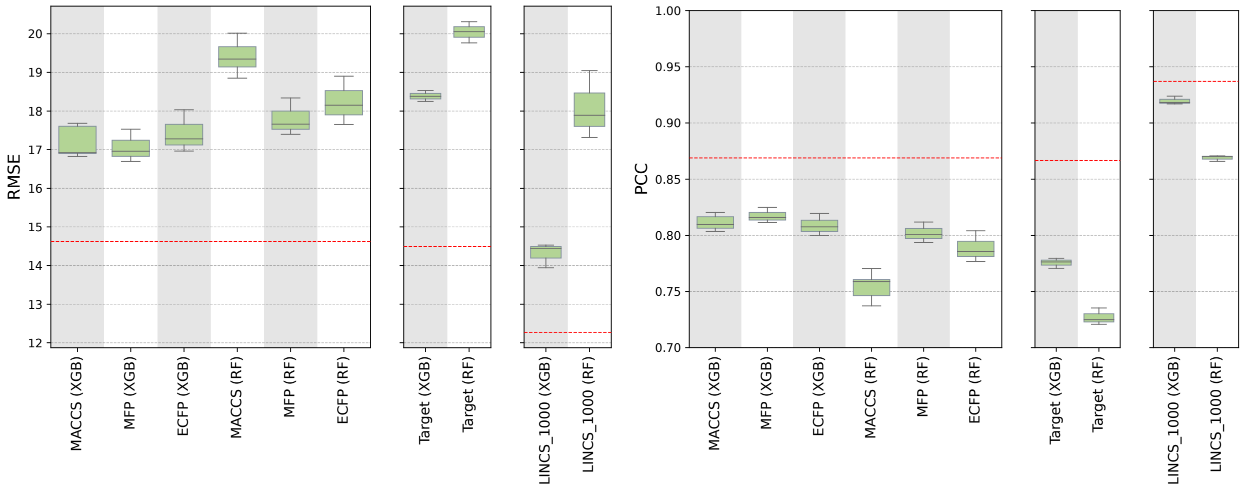

Supplementary Figure 3: **Evaluation of XGB and RF models in predicting S_{mean} scores on DrugComb dataset using leave drug pair split.** The x-axis represents the models, the y-axis displays RMSE (left panel) and PCC (right) between true and predicted scores by each model across independent runs. The red dotted line indicates the median performance of the baseline model.

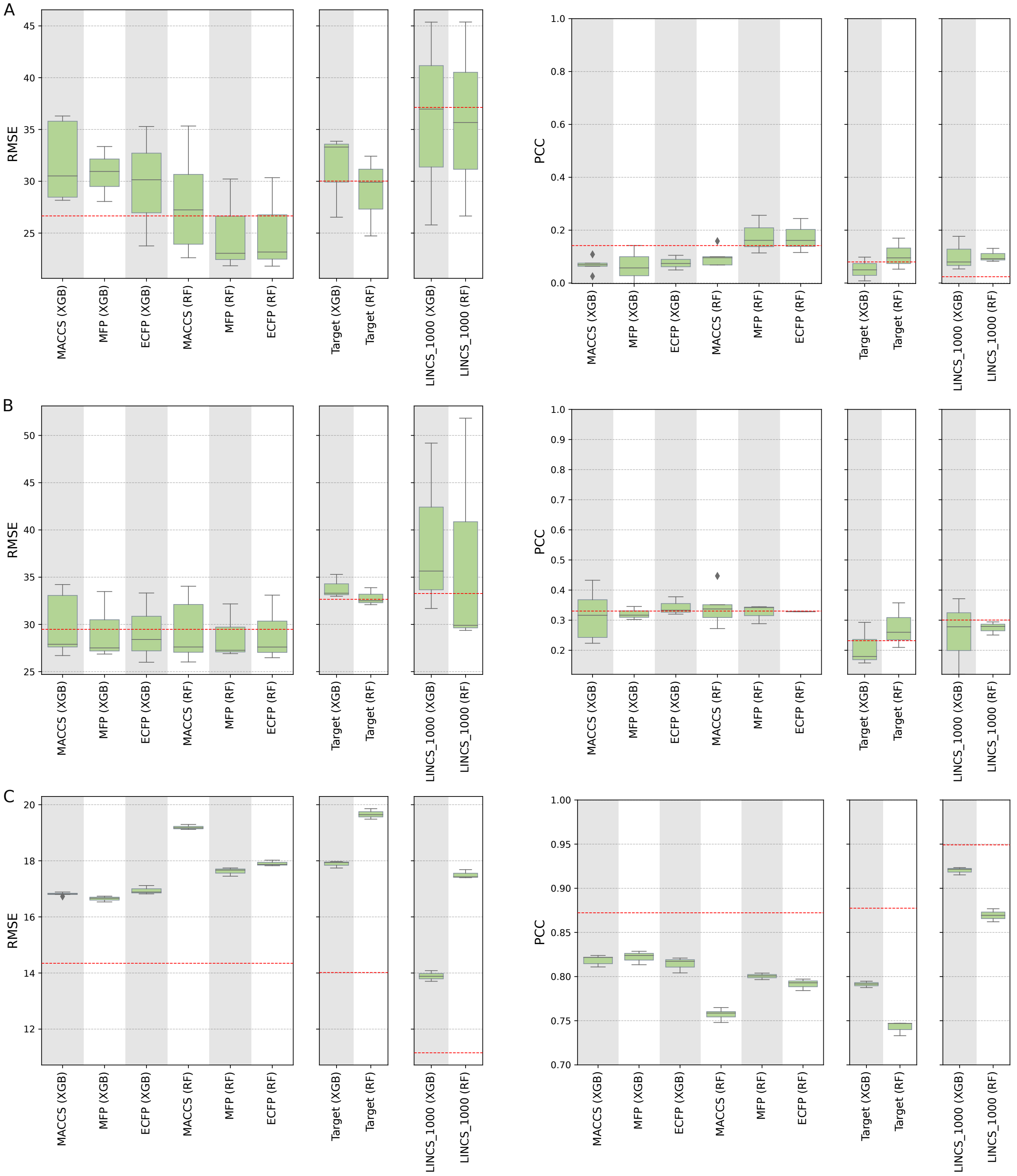

Supplementary Figure 4: **Evaluation of XGB and RF models in predicting S_{mean} scores on DrugComb dataset A.** leave durg split **B.** leave cell line split **C.** leave triplet split. The x-axis represents the models, the y-axis displays RMSE (left panel) and PCC (right) between true and predicted scores by each model across independent runs. The red dotted line indicates the median performance of the baseline model.

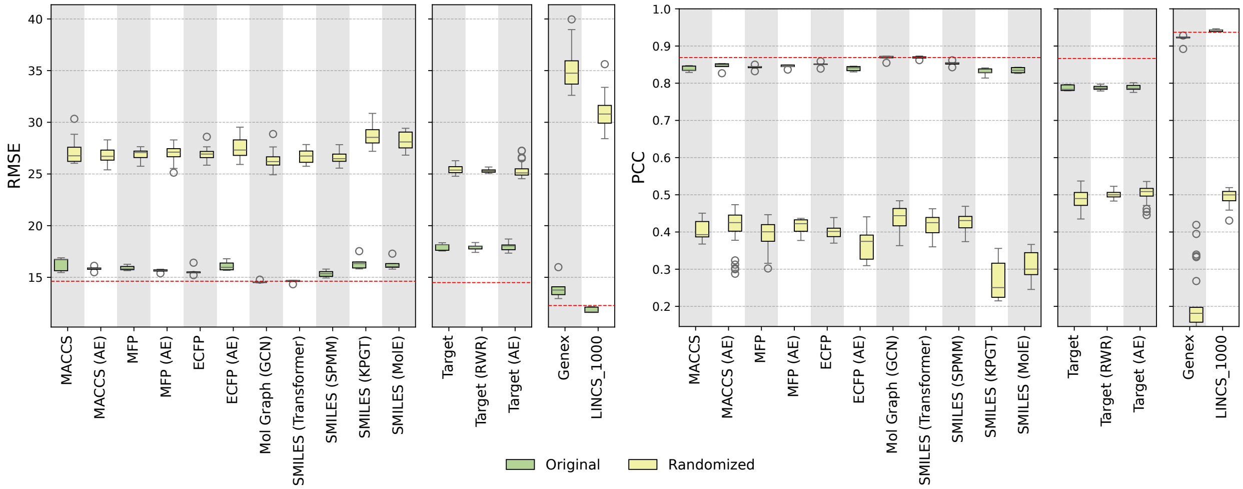

Supplementary Figure 5: **Performance comparison between models with original vs. randomized score using leave drug pair split.** The x-axis represents the models and the y-axis the RMSE or PCC. The red dotted line indicates the median performance of the baseline model.

1.7 Performance of the Models with Randomized Score

For each cell line, we retained all the training triplets but permuted their associated synergy scores. We executed these steps separately for triplets with positive scores and those with negative scores. In other words, we fixed the drug and cell line combinations but shuffled their synergy scores. We then trained the models on these triplets with randomized scores. Under this setup, model performance dropped substantially: the SMILES-based, target-based, and gene expression-based models retained at most 50%, 53%, and 65% of their original performance, respectively (Supplementary Figure 5). This pronounced decline confirms that the strong performance of models trained on strength-preserving rewired networks, retaining up to 99% for both SMILES-based and target-based models, and 98% for gene expression-based models, cannot be attributed to overlapping triplets between the original and rewired datasets.

1.8 Evaluation of Models on Triplets with Extreme Synergy Scores

We evaluated the models on triplets with extreme synergy values from the processed dataset (Supplementary Note 1.2). This allowed us to observe the performance of models when trained and tested on triplets with strong synergy or antagonism. Treating the triplets with positive and negative synergy scores separately, first, we retained the triplets with at least the median synergy score for positive triplets and at most the median value for negative triplets. With this filtering step, SMILES-based subset contained $52,861$ triplets, $2,220$ drugs, and 9 cell lines; the target-based subset included $26,928$ triplets, 275 drugs, and 13 cell lines; and the gene expression-based subset comprised $52,048$ triplets, 194 drugs, and 15 cell lines.

Across four data splitting strategies: leave triplet, leave drug pair, leave drug, and leave cell line, no model significantly outperformed the baseline (adjusted p-value> 0.25; one-sided Mann-Whitney U test with Benjamini-Hochberg correction) (leave triplet split and leave drug pair split in Supplementary Figure 6 and leave drug split and leave cell line split in Supplementary Figure 7).

We also retained triplets above the 75th percentile score for positive triplets and below the 25th percentile for negative triplets. With this filtering step, SMILES-based subset contained $26,431$ triplets, 674 drugs, and 9 cell lines; the target-based subset included $13,463$ triplets, 272 drugs, and 13 cell lines; and the gene expression-based subset comprised $26,025$ triplets, 190 drugs, and 15 cell lines. Again, across four data splitting strategies: leave triplet, leave drug pair, leave drug, and leave cell line, no model significantly outperformed the baseline (adjusted p-value> 0.57; one-sided Mann-Whitney U test with Benjamini-Hochberg correction) (leave triplet split and leave drug pair split in Supplementary Figure 8 and leave drug split and leave cell line split in Supplementary Figure 9).

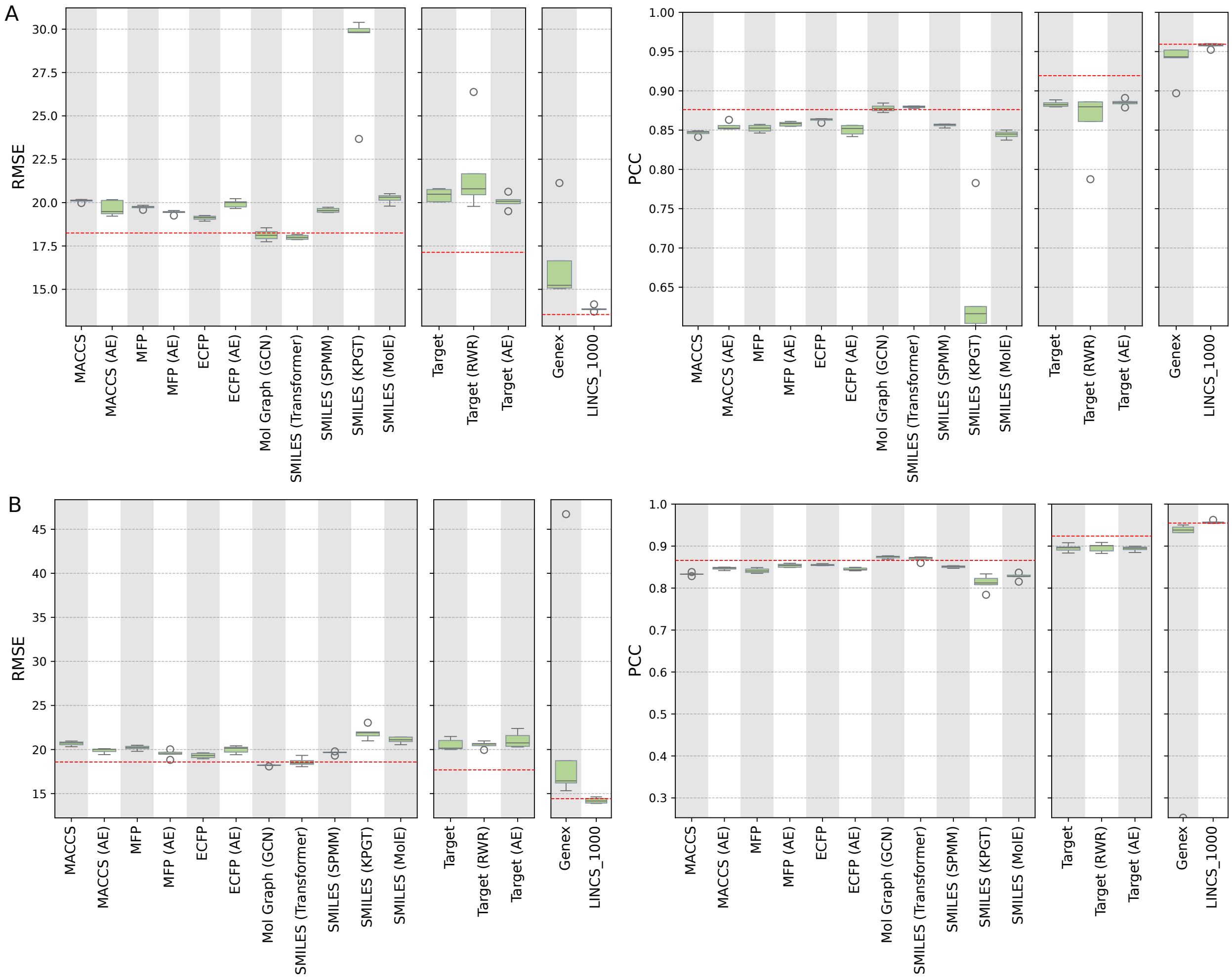

Supplementary Figure 6: **Evaluation of models in predicting S_{mean} score on extreme subset retaining triplets with at least (among positive triplets) and at most (among negative triplets) median synergy score. A.** leave triplet split **B.** leave drug pair split. The x-axis represents the models, the y-axis displays RMSE (left panel) and PCC (right) between true and predicted scores by each model across independent runs. The red dotted line indicates the median performance of the baseline model.

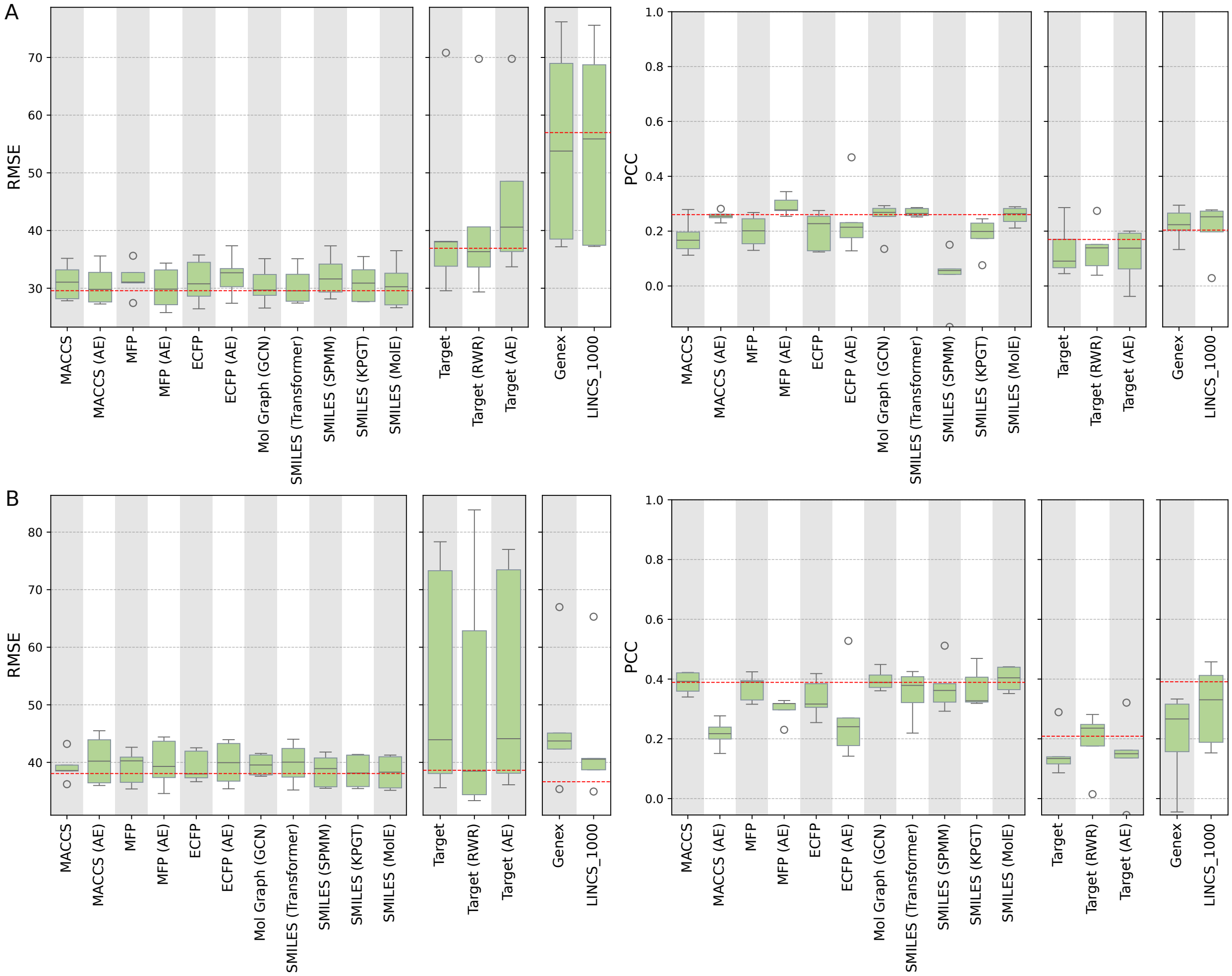

Supplementary Figure 7: **Evaluation of models in predicting S_{mean} score on extreme subset retaining triplets with at least (among positive triplets) and at most (among negative triplets) median synergy score. A.** leave durg split **B.** leave cell line split. The x-axis represents the models, the y-axis displays RMSE (left panel) and PCC (right) between true and predicted scores by each model across independent runs. The red dotted line indicates the median performance of the baseline model.

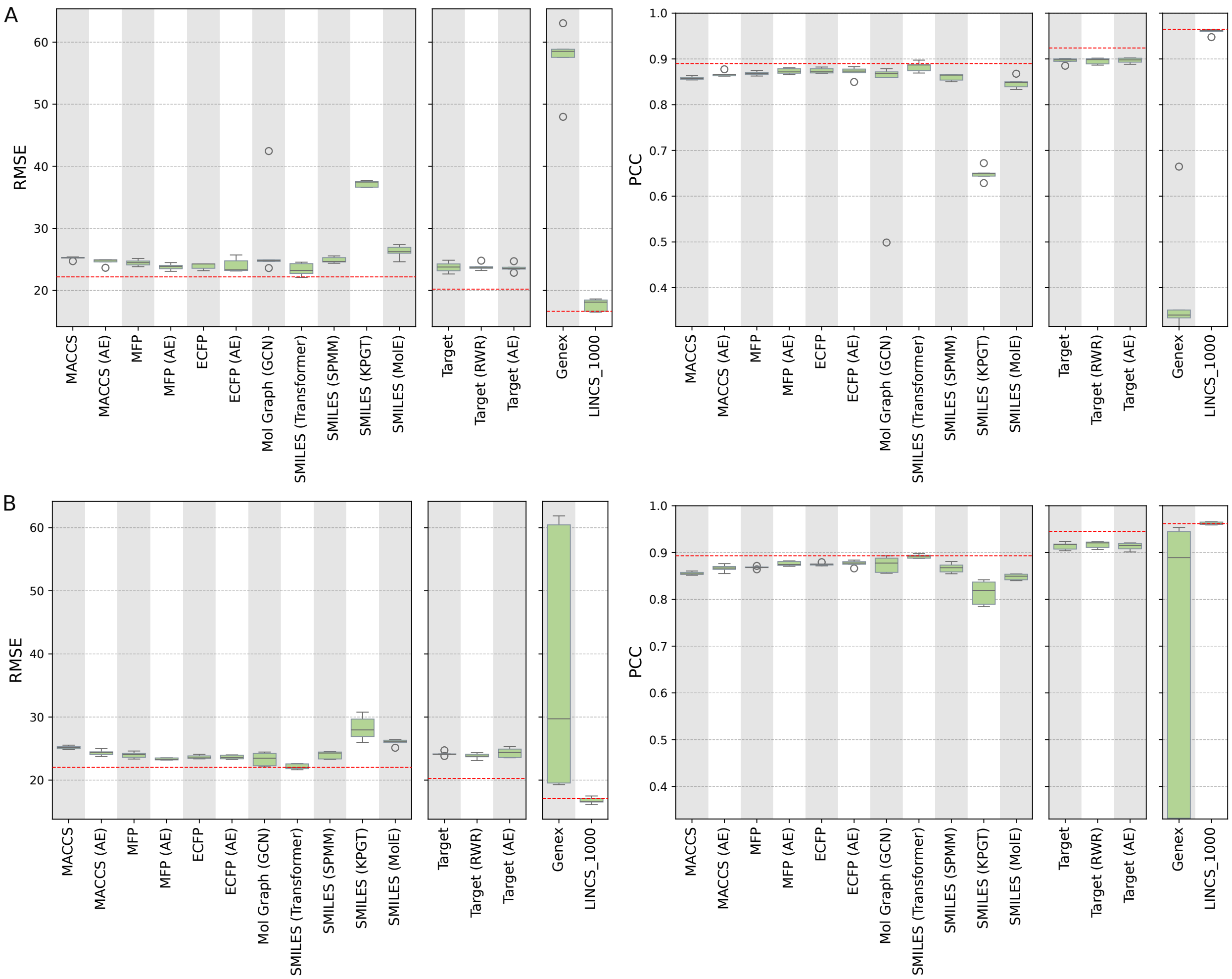

Supplementary Figure 8: **Evaluation of models in predicting S_{mean} score on extreme subset retaining triplets with at least (among positive triplets) 75th and at most (among negative triplets) 25th percentile of synergy score. A.** leave triplet split **B.** leave drug pair split. The x-axis represents the models, the y-axis displays RMSE (left panel) and PCC (right) between true and predicted scores by each model across independent runs. The red dotted line indicates the median performance of the baseline model.

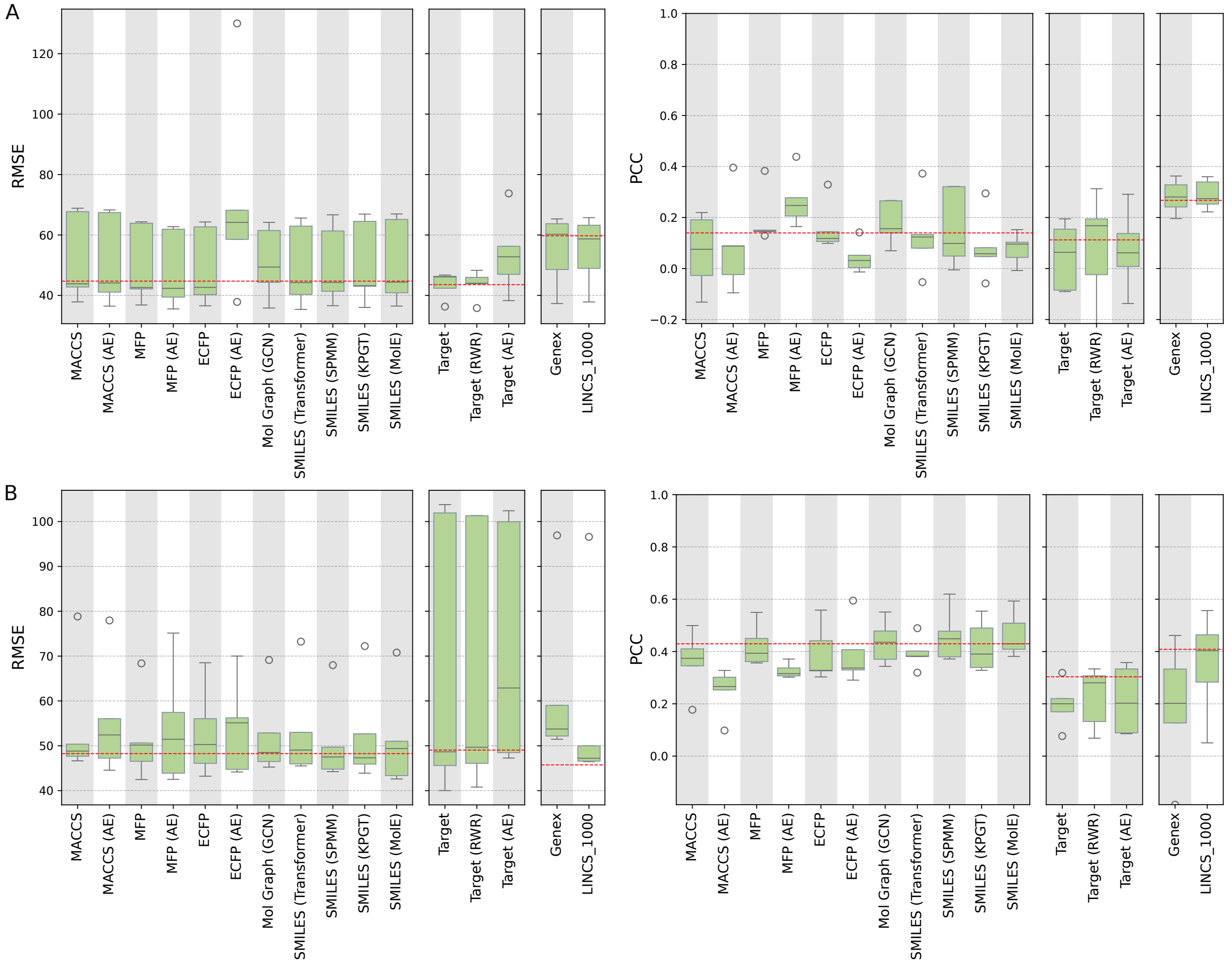

Supplementary Figure 9: **Evaluation of models in predicting S_{mean} score on extreme subset retaining triplets with at least (among positive triplets) 75th and at most (among negative triplets) 25th percentile of synergy score. A.** leave durg split **B.** leave cell line split. The x-axis represents the models, the y-axis displays RMSE (left panel) and PCC (right) between true and predicted scores by each model across independent runs. The red dotted line indicates the median performance of the baseline model.

1.9 Dataset Statistics: Loewe Score

We applied the data processing pipeline for S_{mean} to generate triplets with Loewe synergy scores. Since some triplets in DrugComb did not have both scores, the set of triplets with Loewe scores differed slightly from the one obtained for S_{mean}.

The final datasets consisted of $105,546$, $53,725$, and $103,921$ triplets; $2,582$, 275, and 194 unique drugs; and 9, 13, and 15 cell lines, respectively, for the SMILES-based, target-based, and gene expression-based subsets (Supplementary Figure 10).

In the SMILES-based subset, the cell line kbm7 was the most represented, appearing in $38,227$ triplets (36% of the subset), while wm115 had the fewest, with $5,359$ triplets (5%). This subset showed a mean synergy score of -5.17 with a standard deviation of 16.84.

In the target-based subset, kbm7 again had the highest representation, contributing $15,225$ triplets (28%), whereas ipc298 appeared least frequently with $2,775$ triplets (5%). The average synergy score in this subset was -3.91, with a standard deviation of 16.01.

In the gene expression-based subset, skmel28 contributed the most triplets $(9,604; 9\%)$ and ipc298 the least $(5,356; 5\%)$. This subset had an average synergy score of -6.09 and a standard deviation of 15.53.

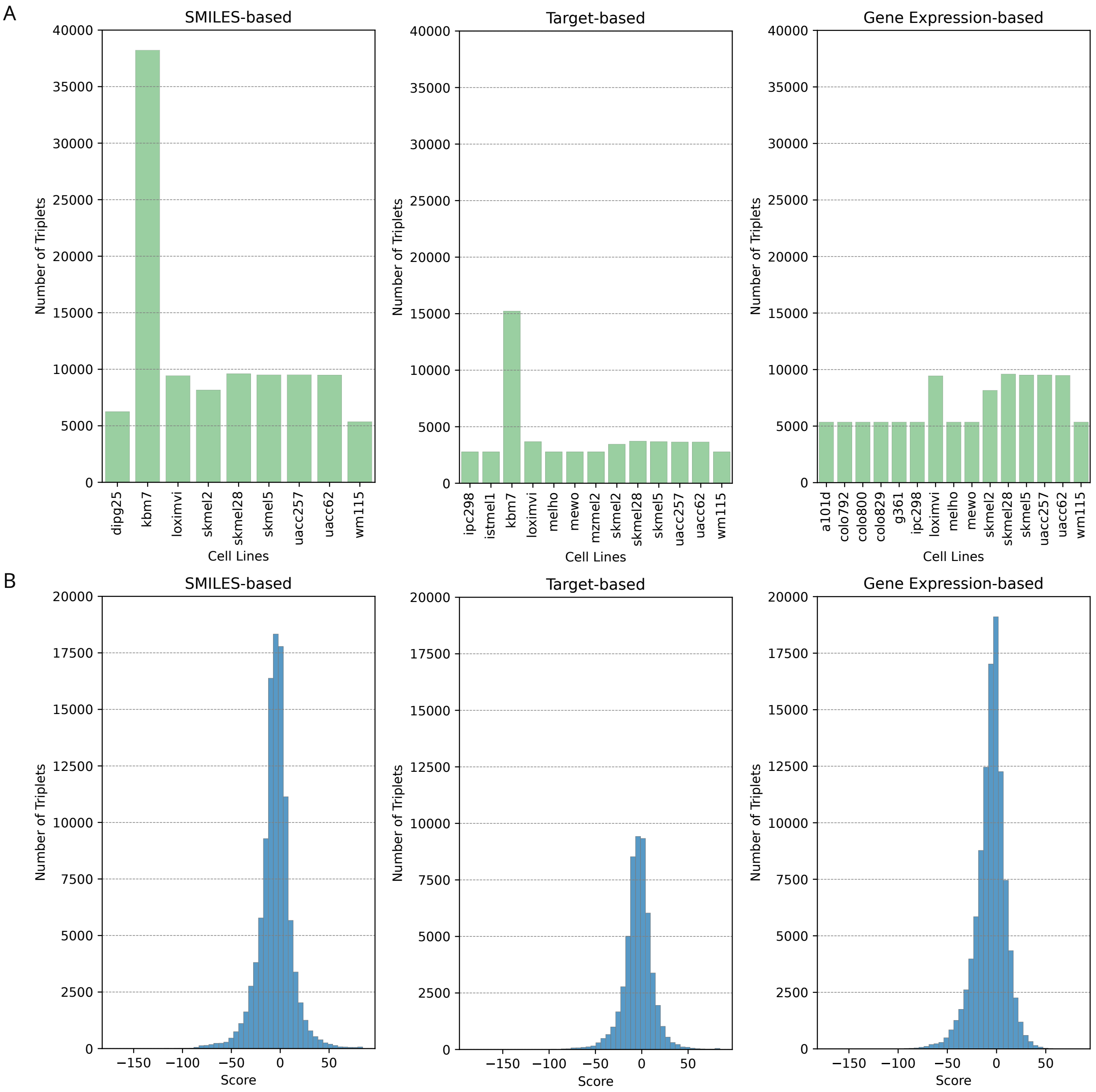

Supplementary Figure 10: **Distribution of Loewe score in the DrugComb dataset**. **A.** The x-axis shows the names of cell lines, and the y-axis indicates the number of drug-drug-cell line triplets associated with each cell line. **B.** The x-axis displays binned Loewe synergy scores, and the y-axis shows the number of triplets falling within each score bin.

1.10 Performance of the Models Predicting Loewe Score

We used the same training and evaluation pipeline as for S_{mean} to predict Loewe synergy scores. Across three data splitting strategies: leave drug pair, leave drug, and leave cell line, no model significantly outperformed the baseline using one-hot encoded drug and cell line features (adjusted p-value> 0.1; one-sided Mann-Whitney U test with Benjamini-Hochberg correction) (Supplementary Figure 11). This outcome mirrors our findings for S_{mean} prediction.

Interestingly, under the leave-drug split, SMILES-based models achieved RMSE values ranging from 16.59 to 19.42, and target-based models from 16.98 to 20.97, noticeably lower than the best RMSE of 27.52 observed for S_{mean} in the same models. However, the corresponding PCC values remained low (between 0.05 and 0.15 for SMILES-based, between 0.03 and 0.09 for target-based models), indicating that the lower RMSE does not reflect better generalization.

Upon examining the data distribution, we found that the standard deviation of Loewe scores ranged from 15.53 to 16.84 (Supplementary Note 1.9), comparable to the achieved RMSE. In contrast, S_{mean} scores exhibited much higher variability (standard deviation between 29.23 and 36.25 (Supplementary Note 1.2). Given a dataset with a mean score of a and standard deviation b, a simple model predicting the value a as the score for every sample will yield an RMSE close to b. Our models likely behave similarly. The low PCC further supports this hypothesis: although the models reduce RMSE by predicting values that are close to the average, they fail to preserve the rank order of the true synergy scores, thus resulting in a low value of PCC.

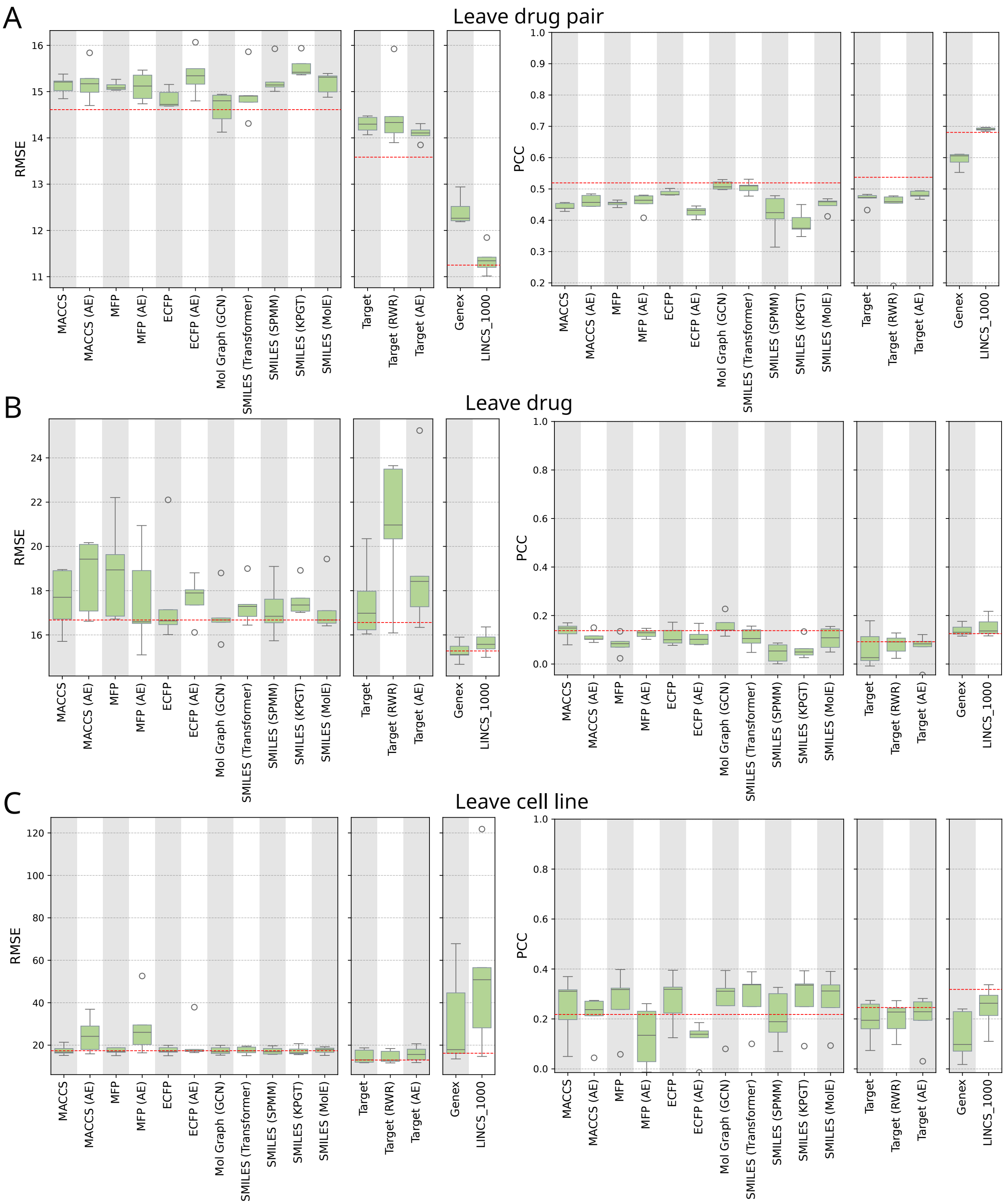

Supplementary Figure 11: **Evaluation of models in predicting Loewe synergy score using A.** leave drug pair split **B.** leave drug split **C.** leave cell line split. The x-axis represents the models, the y-axis displays RMSE (left panel) and PCC (right) between true and predicted scores by each model across five independent runs. The red dotted line indicates the median performance of the baseline model.

1.11 Performance of Models Predicting Loewe Scores on the O'Neil Dataset

The DrugComb dataset includes synergy scores from multiple publications. Since there may be batch effects between different studies, we also evaluated SynVerse on a single dataset. For this purpose, we selected the O'Neil dataset, one of the first large-scale synergy studies, thereby using only triplets from a single experiment ([9]). This initial dataset contained $22,737$ unique triplets across 38 drugs and 39 cell lines. The SMILES-based subset contained all the triplets from the initial dataset. However, the gene expression-based subset comprised $16,907$ triplets with 38 drugs and 29 cell lines. We did not run the target-based models, as we had the target data available from TTD ([10]) only for four drugs. We used Loewe scores for this dataset.

Across four data splitting strategies: leave triplet, leave drug pair, leave drug, and leave cell line, no model significantly outperformed the baseline using one-hot encoded drug and cell line features (adjusted p-value> 0.28; one-sided Mann-Whitney U test with Benjamini-Hochberg correction) (leave triplet split and leave drug pair split in Supplementary Figure 12 and leave drug split and leave cell line split in Supplementary Figure 13). This outcome mirrors our findings for model performance on the DrugComb dataset.

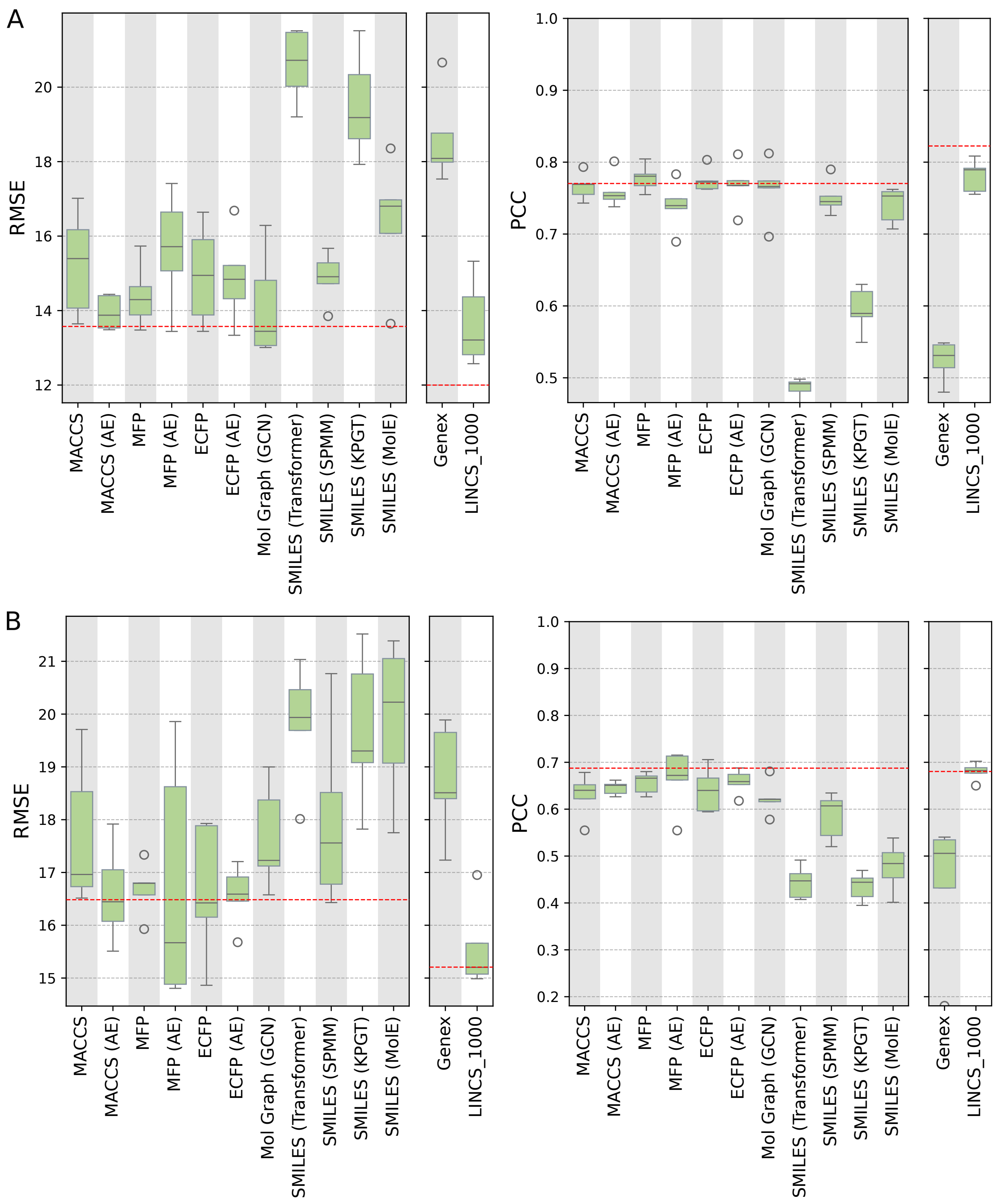

Supplementary Figure 12: **Evaluation of models in predicting Loewe synergy score on O'Neil dataset using A.** leave triplet split **B.** leave drug pair split. The x-axis represents the models, the y-axis displays RMSE (left panel) and PCC (right) between true and predicted scores by each model across five independent runs. The red dotted line indicates the median performance of the baseline model.

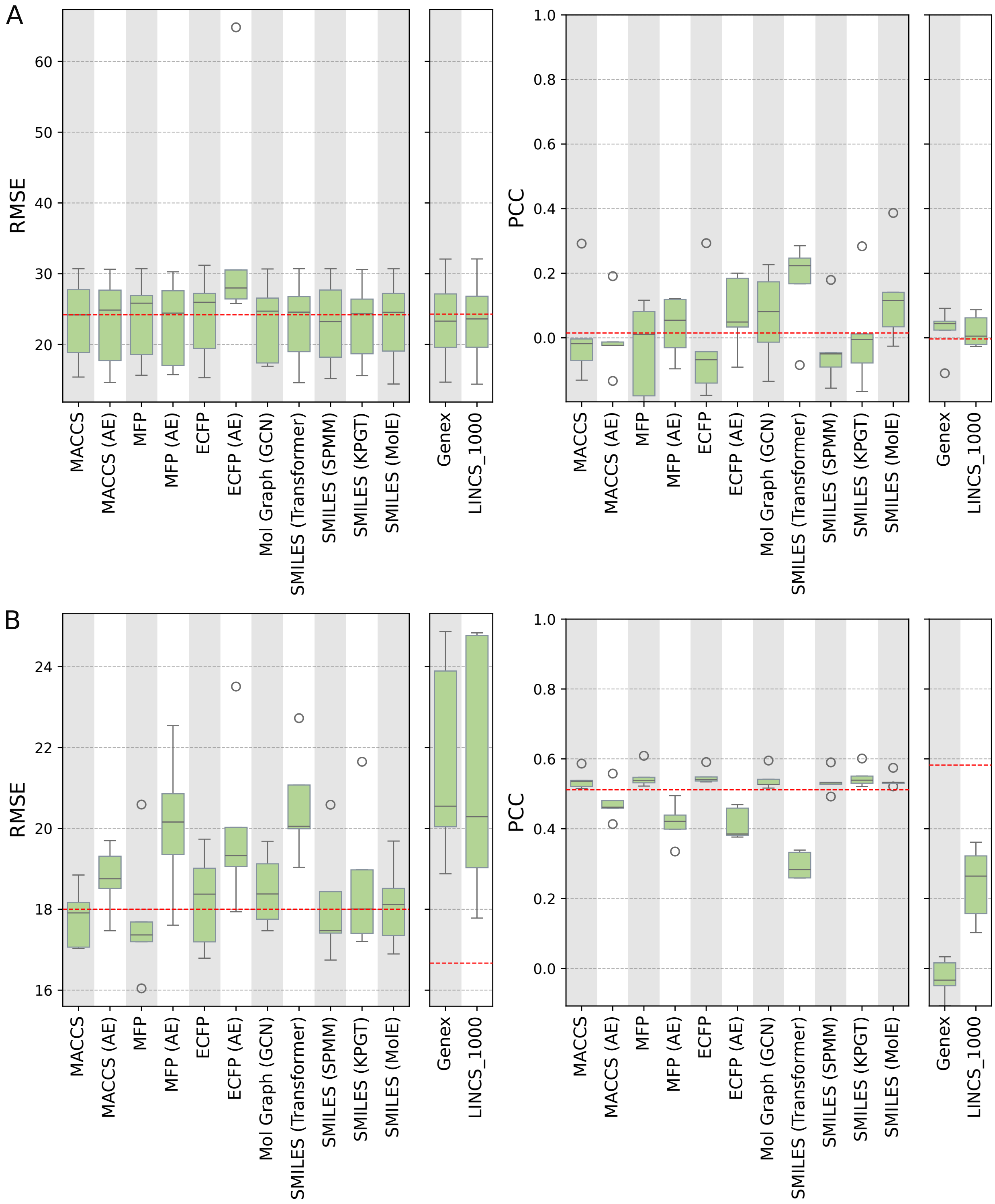

Supplementary Figure 13: **Evaluation of models in predicting Loewe synergy score on the O'Neil dataset using A.** leave drug split **B.** leave cell line split. The x-axis represents the models, the y-axis displays RMSE (left panel) and PCC (right) between true and predicted scores by each model across five independent runs. The red dotted line indicates the median performance of the baseline model.

1.12 Preprocessing Module in SynVerse

We provide the following options for preprocessing in SynVerse: **Autoencoder.** Many of the extracted features, such as drug target profiles and molecular fingerprints, are sparse binary vectors. Hence, we provide a preprocessing step involving an autoencoder to generate a low-dimensional, dense feature representation. After training, we extracted the embedding from the bottleneck layer (the layer with the lowest dimension) of the autoencoder.
RWR on PPI. Given the proteins targeted by a drug and a protein-protein interaction network, we compute a numerical drug target profile using the random walk with restart (RWR) [11] algorithm. By treating the drug targets as restart nodes, we calculated the probabilities of a random walker reaching each node in the network at steady state. We used the corresponding vector of node probabilities as the profile of the drug. This approach is motivated by the need to capture the drug's impact on non-target proteins.
Pretrained molecular foundation models. Recent advancements in molecular foundation models, pretrained on extensive molecular datasets, have demonstrated their capability to learn robust, generalizable, and informative representations of drugs. We used three such pretrained foundation models to investigate whether their embeddings can enhance synergy prediction: KPGT [12], SPMM [13], and MolE [14].

Knowledge-guided Pre-training of Graph Transformer (KPGT) [12] is a self-supervised learning framework to provide generalizable and robust molecular representations. KPGT combines the Line Graph Transformer (LiGhT), tailored for molecular graph structures, with a knowledge-guided pre-training strategy to capture both structural and semantic knowledge. Pre-trained on a large dataset of around 2 million molecules, KPGT demonstrated superior performance in molecular property prediction and practical applicability in drug discovery.

Building on the strengths of multimodal learning, Structure-Property Multi-Modal foundation model (SPMM) [13] integrates molecular structures and biochemical properties using a Transformer architecture. SPMM extracts intramodal features and performs intermodal fusion via self-attention and cross-attention mechanisms, respectively. SPMM is pretrained on 50 million SMILES representations from PubChem [15] and 53 molecular properties calculated with RDKit [16]. SPMM has demonstrated its ability to generalize as a foundation model by performing well on bidirectional tasks such as property prediction (SMILES-to-properties) and property-conditioned molecule generation (properties-to-SMILES) and unimodal tasks, including molecule classification and reaction prediction.

MolE (Molecular Embeddings) [14] is another foundation model with a transformer-based architecture. MolE is trained on molecular graphs from approximately 842 million molecules using a self-supervised pretraining strategy that enabled the model to learn by predicting the environment (i.e., the atom type and connectivity of all neighboring atoms) around each atom. It adapted the disentangled attention mechanism from DeBERTa ([17]) to incorporate relative positional information between atoms within a molecular graph. These innovations enabled MolE to produce embeddings that were well-suited for various downstream tasks. However, since the version of MolE pretrained on 842 million molecules was unavailable, we utilized a smaller model provided by the authors, which was pretrained on the GuacaMol dataset [18] comprising approximately 1.2 million compounds.

1.13 Data Splitting Strategies

We provide the following four data splitting strategies in SynVerse:

1. **Leave triplet.** We split the drug-drug-cell line triplets uniformly at random into train (80%) and test (20%) sets. This approach evaluates how a model performs when it sees a new triplet.

2. **Leave drug pair.** We considered the set of unique drug pairs (ignoring the cell lines in a triplet) and split them uniformly at random into two sets T_1 (80% of drug pairs) and T_2 (20% of drug pairs). We formed the training (respectively, test) set by including all triplets that involved a drug pair in T_1 (respectively, T_2). Hence, we excluded any drug pair present in the test set from the training set, i.e., if a triplet (d_i, d_j, c_k) appeared in the test set, all triplets involving the drug pair (d_i, d_j) were also in the test set. Unlike the "leave triplet" split, this strategy ensures that no drug pair appears in both the training and test sets.

3. **Leave drug.** Analogous to "leave drug pair", we partitioned the set of unique drugs into two sets T_1 and T_2. We formed the training (respectively, test) set by including all triplets where both drugs were present in T_1 (respectively, T_2). Consequently, no drug present in the test set appeared in the training set, i.e., if a triplet (d_i, d_j, c_k) was present in the test set, then all triplets with either d_i or d_j were also in the test set. Note that this split excluded triplets in which one drug appeared in T_1 and the other in T_2. This strategy allows us to assess a model's capability in predicting synergy when encountering a novel drug.

4. **Leave cell line.** Here, we split the triplet by cell lines. Hence, any cell line present in the test set was not present in the training set, i.e., if (d_i, d_j, c_k) was present in the test set, then all triplets from cell line c_k were also in the test set. This strategy evaluates a model's predictive performance on novel cell lines.

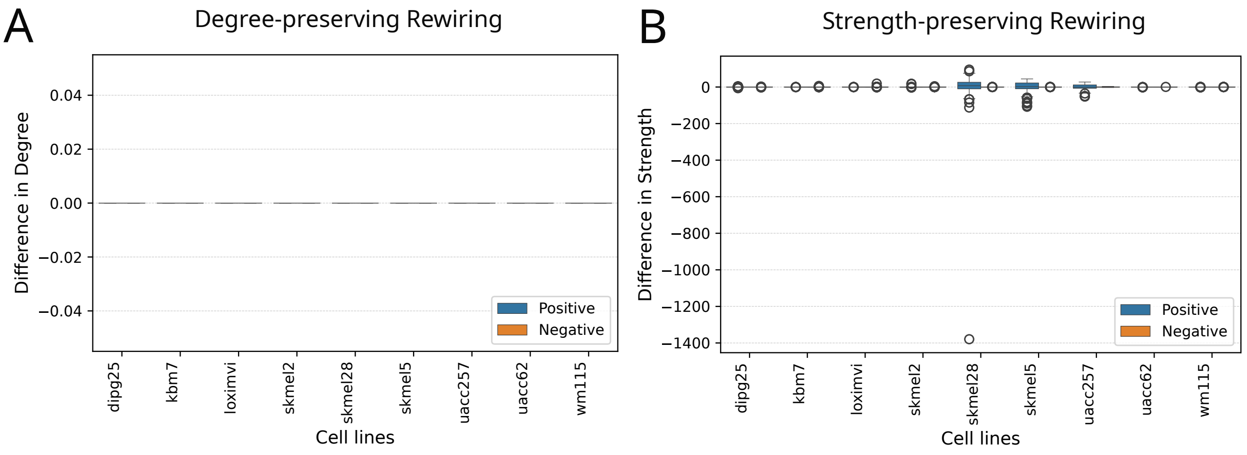

Supplementary Figure 14: **Box plot of differences in degree and strength sequence between original and rewired network.** **A.** The x-axis represents the cell lines. The y-axis represents the difference in degree (of each node) between the original and network rewired using the Maslov-Sneppen method. **B.** The x-axis represents the cell lines. The y-axis represents the difference in strength (of each node) between the original and network rewired using the simulated annealing-based method.

1.14 Network rewiring for network-based ablation study

We used the Maslov-Sneppen [19] randomization method provided in the Brain Connectivity Toolbox (`https://github.com/aestrivex/bctpy`) to rewire the original undirected network while preserving the degree sequence (Supplementary Figure 14A). For strength preserving randomization, we used a simulated annealing-based method [20] (Supplementary Figure 14B). For all these methods, we used approximately 10 swaps per edge.

References

[1] S. Falkner, A. Klein, and F. Hutter, "BOHB: Robust and efficient hyperparameter optimization at scale," in *International Conference on Machine Learning*, pp. 1437–1446, PMLR, 2018.

[2] L. Li, K. Jamieson, G. DeSalvo, A. Rostamizadeh, and A. Talwalkar, "Hyperband: A novel bandit-based approach to hyperparameter optimization," *Journal of Machine Learning Research*, vol. 18, no. 185, pp. 1–52, 2018.

[3] J. Bergstra, R. Bardenet, Y. Bengio, and B. Kégl, "Algorithms for hyper-parameter optimization," *Advances in Neural Information Processing Systems*, vol. 24, 2011.

[4] M. R. El Khili, S. A. Memon, and A. Emad, "MARSY: a multitask deep-learning framework for prediction of drug combination synergy scores," *Bioinformatics*, vol. 39, no. 4, p. btad177, 2023.

[5] Y. Guo, H. Hu, W. Chen, H. Yin, J. Wu, C.-Y. Hsieh, Q. He, and J. Cao, "SynergyX: a multi-modality mutual attention network for interpretable drug synergy prediction," *Briefings in Bioinformatics*, vol. 25, no. 2, p. bbae015, 2024.

[6] T. Chen and C. Guestrin, "Xgboost: A scalable tree boosting system," in *Proceedings of the 22nd acm sigkdd international conference on knowledge discovery and data mining*, pp. 785–794, 2016.

[7] L. Breiman, "Random forests," *Machine learning*, vol. 45, no. 1, pp. 5–32, 2001.

[8] F. Pedregosa, G. Varoquaux, A. Gramfort, V. Michel, B. Thirion, O. Grisel, M. Blondel, P. Prettenhofer, R. Weiss, V. Dubourg, *et al.*, "Scikit-learn: Machine learning in Python," *the Journal of machine Learning research*, vol. 12, pp. 2825–2830, 2011.

[9] J. O'Neil, Y. Benita, I. Feldman, M. Chenard, B. Roberts, Y. Liu, J. Li, A. Kral, S. Lejnine, A. Loboda, *et al.*, "An unbiased oncology compound screen to identify novel combination strategies," *Molecular Cancer Therapeutics*, vol. 15, no. 6, pp. 1155–1162, 2016.

[10] Y. Zhou, Y. Zhang, D. Zhao, X. Yu, X. Shen, Y. Zhou, S. Wang, Y. Qiu, Y. Chen, and F. Zhu, "TTD: Therapeutic Target Database describing target druggability information," *Nucleic Acids Research*, vol. 52, no. D1, pp. D1465–D1477, 2024.

[11] L. Page, S. Brin, R. Motwani, and T. Winograd, "The PageRank citation ranking: Bringing order to the web.," tech. rep., Stanford Infolab, 1999.

[12] H. Li, R. Zhang, Y. Min, D. Ma, D. Zhao, and J. Zeng, "A knowledge-guided pre-training framework for improving molecular representation learning," *Nature Communications*, vol. 14, no. 1, p. 7568, 2023.

[13] J. Chang and J. C. Ye, "Bidirectional generation of structure and properties through a single molecular foundation model," *Nature Communications*, vol. 15, no. 1, p. 2323, 2024.

[14] O. Méndez-Lucio, C. A. Nicolaou, and B. Earnshaw, "MolE: a foundation model for molecular graphs using disentangled attention," *Nature Communications*, vol. 15, no. 1, p. 9431, 2024.

[15] S. Kim, J. Chen, T. Cheng, A. Gindulyte, J. He, S. He, Q. Li, B. A. Shoemaker, P. A. Thiessen, B. Yu, *et al.*, "PubChem in 2021: new data content and improved web interfaces," *Nucleic Acids Research*, vol. 49, no. D1, pp. D1388–D1395, 2021.

[16] G. Landrum, "RDKit: Open-source cheminformatics. 2006," *Google Scholar*, 2006.

[17] P. He, X. Liu, J. Gao, and W. Chen, "DeBERTa: Decoding-enhanced bert with disentangled attention," *arXiv preprint arXiv:2006.03654*, 2020.

[18] N. Brown, M. Fiscato, M. H. Segler, and A. C. Vaucher, "GuacaMol: benchmarking models for de novo molecular design," *Journal of Chemical Information and Modeling*, vol. 59, no. 3, pp. 1096–1108, 2019.

[19] S. Maslov and K. Sneppen, "Specificity and stability in topology of protein networks," *Science*, vol. 296, no. 5569, pp. 910–913, 2002.

[20] F. Milisav, V. Bazinet, R. F. Betzel, and B. Misic, "A simulated annealing algorithm for randomizing weighted networks," *Nature Computational Science*, vol. 5, no. 1, pp. 48–64, 2025.
